# Rice Na^+^-Permeable Transporter OsHAK12 Mediates Shoots Na^+^ Exclusion in Response to Salt Stress

**DOI:** 10.3389/fpls.2021.771746

**Published:** 2021-12-07

**Authors:** Linan Zhang, Xiangyu Sun, Yanfang Li, Xuan Luo, Shaowen Song, Yan Chen, Xiaohui Wang, Dandan Mao, Liangbi Chen, Sheng Luan

**Affiliations:** ^1^Hunan Province Key Laboratory of Crop Sterile Germplasm Resource Innovation and Application, College of Life Sciences, Hunan Normal University, Changsha, China; ^2^Hunan Institute of Microbiology, Changsha, China; ^3^Department of Plant and Microbial Biology, University of California, Berkeley, Berkeley, CA, United States

**Keywords:** rice, OsHAK12, shoot Na^+^ exclusion, retrieving Na^+^ from xylem sap, salt tolerance, Na^+^-permeable transporter

## Abstract

Soil salinity has become a major stress factor that reduces crop productivity worldwide. Sodium (Na^+^) toxicity in a number of crop plants is tightly linked with shoot Na^+^ overaccumulation, thus Na^+^ exclusion from shoot is crucial for salt tolerance in crops. In this study, we identified a member of the high-affinity K^+^ transport family (HAK), OsHAK12, which mediates shoots Na^+^ exclusion in response to salt stress in rice. The *Oshak12* mutants showed sensitivity to salt toxicity and accumulated more Na^+^ in the xylem sap, leading to excessive Na^+^ in the shoots and less Na^+^ in the roots. Unlike typical HAK family transporters that transport K^+^, OsHAK12 is a Na^+^-permeable plasma membrane transporter. In addition, *OsHAK12* was strongly expressed in the root vascular tissues and induced by salt stress. These findings indicate that OsHAK12 mediates Na^+^ exclusion from shoot, possibly by retrieving Na^+^ from xylem vessel thereby reducing Na^+^ content in the shoots. These findings provide a unique function of a rice HAK family member and provide a potential target gene for improving salt tolerance of rice.

## Highlight

- Rice OsHAK12 mediates shoots Na^+^ exclusion under salt stress.

## Introduction

Soil salinity represents a primary hazard to crop productivity (Munns and Tester, 2008; Ismail and Horie, 2017; Munns et al., 2020). Cellular sodium ion (Na^+^) toxicity is the dominant ion toxicity among salinity stress factors, leading to the inhibition of a series of physiological and biochemical processes such as photosynthesis, protein synthesis and K^+^ absorption (Tester and Davenport, 2003; Ismail and Horie, 2017; Zelm et al., 2020). To adapt to high-Na^+^ environments, plants utilize various mechanisms to cope with Na^+^ toxicity, including Na^+^ efflux from roots to the rhizosphere, Na^+^ sequestration in vacuoles, and Na^+^ recycling in plants through vasculature (Munns and Tester, 2008; Ismail and Horie, 2017; Yang and Guo, 2018a,b; Tian et al., 2021). Understanding and harnessing the mechanisms responsed to salt stress will contribute to breeding salt-tolerant crops, thereby safeguarding global food security.

Under saline conditions, Na^+^ is absorbed by plant roots and further delivered to the shoot through the transpiration flow (Ismail and Horie, 2017; Zelm et al., 2020). Excessive Na^+^ translocation from root to shoot and subsequent Na^+^ accumulation in shoot are harmful to crops, such as reduction of carbon assimilation in photosynthetic tissue (Munns and Tester, 2008; Ismail and Horie, 2017). Thus, mechanisms for Na^+^ exclusion from shoot are pivotal for the adaptation of plants in high-Na^+^ environments. Previous studies showed that Na^+^ retrieving from xylem sap in the root is an essential physiological strategy to achieve low shoots Na^+^ concentrations during salt toxicity (Ismail and Horie, 2017; Zelm et al., 2020; Tian et al., 2021). This process is mediated by a number of ion transporters (Horie et al., 2009; Zelm et al., 2020). The *HKT1* family genes which encodes Na^+^-selective transporters have been demonstrated to play crucial roles in this regulatory process. For example, *Arabidopsis HKT1* is strongly expressed in root stelar cells and functions in shoots Na^+^ exclusion by retrieving Na^+^ from the xylem sap in the root (Sunarpi et al., 2005; Davenport et al., 2007; Møller et al., 2009). In addition, the rice salt-tolerant QTL *SKC1/OsHKT1;5*, the wheat salt-tolerant QTLs *Nax1/TmHKT1;4* and *Nax2/TmHKT1;5*, and the maize salt-tolerant QTL *ZmNC1/ZmHKT1* all encode HKT-type Na^+^ transporters that function similarly to *Arabidopsis* HKT1 (Ren et al., 2005; Huang et al., 2006; Byrt et al., 2007; Munns et al., 2012; Zhang et al., 2018). These studies have showed that Na^+^- permeable HKT1 transporters mediate Na^+^ retrieving from xylem vessels and beneficial for enhancement of salt tolerance. Apart from HKT1 family transporters, it remains largely unknown if other types transporters are also involved in retrieving Na^+^ from xylem vessels.

Rice is a staple food and its growth and productivity are highly susceptible to salt tress (Ren et al., 2005; Ismail and Horie, 2017; Kobayashi et al., 2017). The genomes of the *Nipponbare* rice subspecies encode 27 OsHAK family members, four of which have been shown to mediate rice K^+^/Na^+^ homeostasis during salt stress. For example, OsHAK1, OsHAK5, and OsHAK16 are induced by salt stress and involved in salt tolerance (Yang et al., 2014; Chen et al., 2015; Feng et al., 2019). OsHAK21 is essential to maintain Na^+^/K^+^ homeostasis and promote seed germination and seedling establishment under salinity stress (Shen et al., 2015; He et al., 2019). These studies indicate that root K^+^ uptake mediated by HAK family members has great importance for plant salt tolerance. However, it remains unknown whether rice high-affinity K^+^ transport family (KT/HAK/KUP) members serves as Na^+^ transporters thereby functioning in salt tolerance in plants. When studying the function of OsHAK12 in rice, we found that OsHAK12, like several OsHAK members described above, was involved in salt tolerance as its mutants were salt sensitive. Surprisingly, OsHAK12, unlike previously reported HAK members, failed to transport K^+^ but instead transported Na^+^ as assayed in yeast mutants. Consistent with this transport activity, OsHAK12 apparently served as a Na^+^- permeable transporter that retrieved Na^+^ from xylem back to root tissues and thus protected plants from salt toxicity by excluding Na^+^ from shoots.

## Materials and Methods

### Plant Material and Growth Conditions

Japonica rice cultivar *Nipponbare* (*O. sativa* L.) was used as the wild type in this study, and also used for the generation of all transgenic plant lines. IRRI (International Rice Research Institute) hydroponic solution for rice was conducted as previous method (Li et al., 2014; Wang et al., 2021). The modification of Na^+^ and K^+^ concentrations as indicated in the figure legends.

### Yeast Functional Complementation

The rice genomic cDNA sequences encoding *OsHAK12* was amplified by PCR using the primer pairs listed in Supplementary Table 1. The PCR product was constructed into pYES2 vector (digested with *Hin*dIII and *Xba*I) to generate pYES2-NC-OsHAK12. This construct and the empty vector were transformed into yeast strain K^+^ uptake-deficient CY162 or high-Na^+^ sensitive AXT3K, respectively. The yeast complementation assay were performed as previous methods (Anderson et al., 1992; Quintero et al., 2002).

### qRT-PCR Analysis

Total RNA was isolated from *Nipponbare* rice using the TRIzol reagent (Invitrogen). Real time qRT-PCR analyses were performed as described previously (Livak and Schmittgen, 2001; Wang et al., 2021). All primers used for real time qRT-PCR assay are listed in Supplementary Table 1.

### Histochemical Analysis of GUS Expression

The 2,000-bp fragment located upstream of the *OsHAK12* initiation codon was amplified from *Nipponbare* rice genomic DNA. This amplified promoter fragment was digested with *Eco*RI and *Hin*dIII, then cloned into pCAMBIA1301-GUS vector. The genetic transformation and histochemical analysis of GUS staining in different tissues of rice as described previously (Upadhyaya et al., 2000; Ai et al., 2009; Wang et al., 2021). All primers used for the GUS assay are listed in Supplementary Table 1.

### Subcellular Localization of OsHAK12

The full length cDNA of *OsHAK12* without the stop codon was amplified, after sequence confirmation and digestion with *Xba*I, the amplified DNA fragment was cloned into the binary vector pCAMBIA1390 to generate the 35S:*OsHAK12-GFP* fusion construct. Transient expression of the fusion protein was examined by the confocal laser-scanning microscopy using the LSM880 instrument (Carl Zeiss) as previous methods (Li et al., 2009; Wang et al., 2021). The primers used for the subcellular localization assay are listed in Supplementary Table 1.

### Development of *OsHAK12* CRISPR/Cas9 Knockout Lines

To generate *OsHAK12* knockout plants, the CRISPR/Cas system for targeted genome modification of rice was used (Xie et al., 2014; Usman et al., 2020). A 20-bp sgRNA sequences (GAGAGCTGGACCTCCCTTGG) was cloned into the *pOs-sgRNA* vector, and then subcloned into the Cas9 vector *pYLCRISPR/Cas9Pubi-H* (Supplementary Figure 1). Transgenic plants were obtained and identified as following the procedure (Upadhyaya et al., 2000; Wang et al., 2021). Two T2 generation homozygous mutant lines *Oshak12-1* and *Oshak12-2* were used for further study. The primers used for this assay are listed in Supplementary Table 1.

### Measurement of Chlorophyll and Ion Content Analysis

Measurement of chlorophyll and ion content (Na^+^, K^+^) analysis as previous methods (Porra et al., 1989; Wang et al., 2021). The collected method, Na^+^ and K^+^ concentration in the xylem sap and phloem exudates were determined using inductively coupled plasma/optical emission spectrometry ICP-AES (Varian 715-ES) following the method reported by Tian et al. (2021). Briefly, 5-days-old rice seedlings were cultivated in the solutions for 14 days and then transferred to the hydroponic cultures containing 0 or 100 mM Na^+^ for 2 days. The shoots were cut and then the xylem sap exuding at the cut surfaces was collected for 1 h. The xylem sap exudates were discard at initial half hour and the xylem sap exudates was collected during the first to second hour and the third to fourth hour on the same plants exchanging cotton balls on the same stumps. Then xylem sap was got from the cotton balls by centrifugation. To collect phloem exudates, the shoots were excised from seedling and the cut extremities were immediately dipped in a 15 mM EDTA solution (pH 7.5, K_2_-EDTA buffer was used for Na^+^ content assay and Na_2_-EDTA buffer was used for K^+^ content assay) and incubated in dark for 8 h under 90% humidity condition.

## Results

### Rice *Oshak12* Mutants Are Hypersensitive to Salinity but Not to Low K^+^ Stress

To dissect the functions of rice HAK family members, we generated loss-of-function mutants for each OsHAK transporter using CRISPR-assisted genetic analysis in *Nipponbare* rice background. Here, we focused on the functional identification of OsHAK12. We generated two independent knockout mutants (*Oshak12-1* and *Oshak12-2*) of *OsHAK12*. The *Oshak12-1* and *Oshak12-2* mutants had a 4-bp and 1-bp deletion in the third exon of *LOC_Os08g10550*, respectively, leading to a frameshift mutations at the 192th and 194th amino acids and premature translation termination at 211 and 213 amino acids separately (Supplementary Figure 1). No off-target cleavage was found using the web-based tool CRISPR-P (Liu et al., 2017)^1^.

Previous studies showed that some high affinity K^+^ transporter (HAK) family members responsed to low-K^+^ stress or salt tolerance in plants (Yang et al., 2014; Chen et al., 2015; Shen et al., 2015; Feng et al., 2019; Wang et al., 2021). First, we detected the growth of the *Oshak12* mutants (*Oshak12-1*, *Oshak12-2*) under different K^+^ concentration conditions. We found that the *Oshak12* mutants and the wild-type plants *Nipponbare* (Nip) both grew well with no distinct differences under either K^+^-sufficient (10 mM K^+^) or K^+^-deficient (0.01 mM K^+^) hydroponic solutions (Supplementary Figures 2A–E). In addition to seedling height, fresh weight, we also measured K^+^ content in both roots and shoots and found no differences between wild type and mutants, suggesting that disruption of *OsHAK12* does not affect K^+^ homeostasis in rice at seedling stage. We further found that the grain length, grain width, 1,000-grain weight of the mature grains between wild type and mutants displayed no significant differences (Supplementary Figures 3A,Ba–f). Scanning electron microscopy (SEM) of transverse sections of mature endosperm revealed that the endosperm of *Oshak12* and wild type mature grains both filled with larger, regular, tightly packed starch grains (Supplementary Figure 3C). In addition, no significant differences of pollen viability were observed between the *Oshak12* mutants and the wild type (Supplementary Figures 3D,Ea,b). The above results suggested that disruption of *OsHAK12* does not affect K^+^ homeostasis in rice at reproductive stage.

**FIGURE 1:**
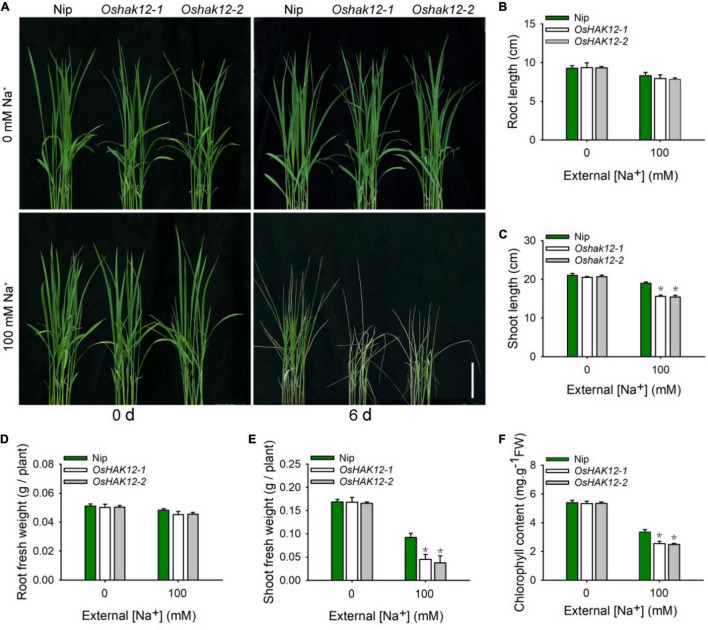
*Oshak12* mutants are more hypersensitive to salt stress. **(A)** *Oshak12* mutants are more hypersensitive to salt toxicity. The seeds of the Nip and *Oshak12* mutants (*Oshak12-1*, *Oshak12-2*) plants germinated in water for 4 days, after transferred to the hydroponic cultures for 14 days, then transferred to the hydroponic cultures containing 0 or 100 mM Na^+^ for 6 days and photographed. The *Oshak12* mutants are more sensitive to salt stress than the Nip. Bars = 6 cm. **(B)** Root length of the Nip and *Oshak12* mutants plants. No significant differences were found between the Nip and *Oshak12* mutants (*n* = 30 for each data point) (*P* > 0.05 by Student’s *t*-test). **(C)** Shoot length of the Nip and *Oshak12* mutants plants. Significant differences were found between the Nip and *Oshak12* mutants (*n* = 30 for each data point) (*P < 0.005 by Student’s *t*-test). **(D)** Root fresh weight of Nip and *Oshak12* mutants plants. No significant differences were found between the Nip and *Oshak12* mutants (*n* = 30 for each data point) (*P* > 0.05 by Student’s *t*-test). **(E)** Shoot fresh weight of Nip and *Oshak12* mutants plants. Significant differences were found between the Nip and *Oshak12* mutants (*n* = 30 for each data point) (*P < 0.005 by Student’s *t*-test). **(F)** ChlorophyII content of Nip and *Oshak12* mutants plants. Significant differences were found between the Nip and *Oshak12* mutants (*n* = 30 for each data point) (*P < 0.005 by Student’s *t*-test). Growth conditions were as described in **(A)**. The experiment was repeated four times with similar results. Data are means of five replicates of one experiment. Asterisks represent significant difference. Error bars represent ± SD.

We then examined the growth of the two independent *Oshak12* mutants under salt stress conditions. We transferred 14-days-old plants of *Oshak12* and wild type grown in hydroponic culture to the same solution plus 100 mM Na^+^ for 6 days and found that the shoots of the *oshak12* mutants displayed more withered and chlorotic phenotype as compared to that of wild type plants. In addition, the *Oshak12* mutants showed decreased shoots growth under salt stress (Figure 1A). The above datas indicated that the *oshak12* mutants were more hypersensitive to salt stress than the wild type plants. To quantify the phenotypes, we further determined the length and fresh weight of roots and shoots separately and measured the chlorophyll contents of the leaves. While the mutants showed similar levels as the wild type in roots, the mutant shoots were much stunted under salt stress as compared to the wild type (Figures 1B–E). Additionally, the chlorophyll contents of *Oshak12* mutants were also lower than that of the wild type plants after NaCl treatment (Figure 1F), consistent with their chlorotic phenotype.

The above results showed that disruption of *OsHAK12* was responsible for the hypersensitivity to salinity stress.

### Expression Pattern and Subcellular Localization of OsHAK12

**FIGURE 2:**
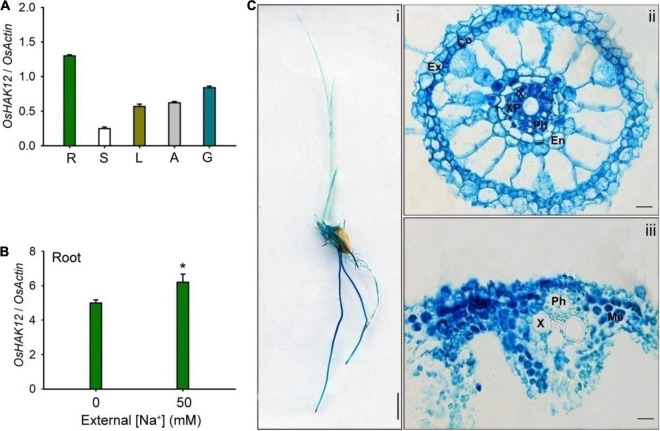
Expression pattern of *OsHAK12*. **(A)** *OsHAK12* mRNA accumulation by real time qRT-PCR analyses in different rice tissues as indicated in this figure. *Nipponbare* rice seedlings were grown in soil for 12 weeks. R, root; S, shoot; L, leaf; A, anther; G, glume. **(B)** The transcriptional expression of *OsHAK12* in rice under different salt concentrations treatment. 3-days-old *Nipponbare* rice seedlings were cultivated in hydroponic culture for 7 days, and then transferred to the culture containing 50 mM Na^+^ for 12 h. Total RNAs were isolated from the rice seedlings, and the mRNA levels of *OsHAK12* were examined by real time qRT-PCR. *OsActin* was used as an internal reference. Significant difference was found between 0 or 50 mM NaCl samples are indicated in rice seedlings (*P < 0.01 by Student’s *t*-test). **(C)** Histochemical analysis of GUS expression for *OsHAK12*. 3-days-old *Nipponbare* rice seedlings were cultivated in hydroponic culture for 4 days, then GUS activities were determined after histochemical staining. Blue indicates GUS activity. (i) GUS staining of 7-days-old plants grown in hydroponic cultures with IRRI solution. (ii) Cross section images of the elongation zone in (i). (iii) Cross section images of the leaf vascular tissue in (i). Ex, exodermis; Co, Cortex; En, endodermis; Ph, phloem; X, xylem; XP, xylem parenchyma; Me, mesophyll cells. Bar in (i) = 1 cm and bars in (i) to (iii) = 100 μm. The experiment was repeated five times with similar results. Data are means of five replicates of one experiment. Asterisks represent significant differences. Error bars represent ± SD.

To understand the physiological role of OsHAK12, we first performed the expression pattern analysis of *OsHAK12* in rice plants. The qRT-PCR analysis showed that *OsHAK12* was expressed strongly in the roots and its lower amounts transcripts were also detected in stems, leaves, anther and glumes (Figure 2A). The expression of *OsHAK12* was up-regulated in root during salt stress (Figure 2B). To detect the expression pattern of *OsHAK12* in more detail, the GUS activity staining of transgenic rice plants harboring the *OsHAK12* promoter-GUS fusion construct was performed. Strong GUS signals were found in the roots of the transgenic rice plants (Figure 2Ci), which was consistent with the qRT-PCR results (Figure 2A). Cross sections of GUS-stained roots showed that *OsHAK12* was expressed almost in all cell types such as root hair, exodermis, cortex and endodermis, especially strongly expressed in vascular tissues (Figure 2Cii). Furthermore, GUS activity was present in mesophyll cells (Figure 2Ciii).

**FIGURE 3:**
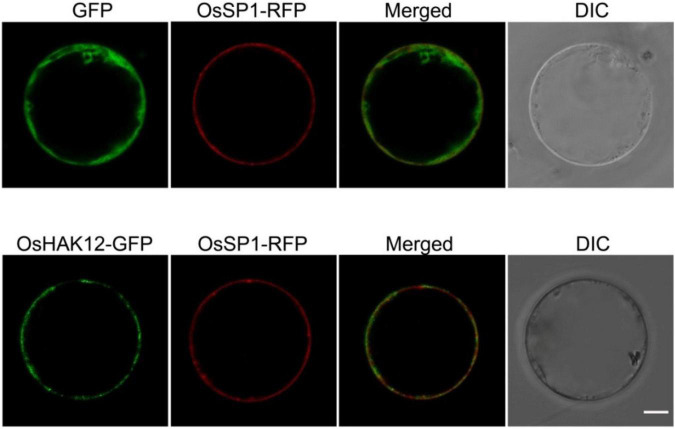
Plasma membrane localization of OsHAK12. GFP, OsHAK12-GFP, and OsSP1-RFP (a plasma membrane localization marker) in rice mesophyll protoplasts. For each localization experiment, ≥ 35 individual cells were analyzed using a Zeiss LSM880 confocal laserscanning microscope (Carl Zeiss). Bar = 10 μm.

Then, we carried out the subcellular localization of OsHAK12 in plant driven by the cauliflower mosaic virus 35S promoter. A green fluorescence protein (GFP) reporter construct was developed to express the OsHAK12-GFP fusion protein, and the same vector expressing GFP only was used as a control. Subsequently, the OsHAK12-GFP fusion construct and the GFP-only control were transformed into the protoplasts of the rice leaf sheaths cells, respectively. GFP-only signal was present mainly in the cytoplasm and nucleus as expected, whereas OsHAK12-GFP fusions was localized at the plasma membrane, as indicated by overlaps between GFP and signals from the known plasma membrane protein fused to red fluorescent protein (SP1-RFP) (Li et al., 2009; Figure 3). Based on these results, we concluded that OsHAK12 is localized to the plasma membrane in rice cells.

### Knockout of *OsHAK12* Leads to Overaccumulation of Shoot Na^+^

Salinity stress generates both osmotic stress and Na^+^ ionic toxicity in plants (Tester and Davenport, 2003; Shen et al., 2015; Zelm et al., 2020). As 100 mM NaCl could cause both osmotic stress and ionic toxicity in plants, we compared the mutant and wild type plants grown under 20% PEG6000 (polyethylene glycol with an average molecular weight of 6,000 Da) that imposed osmotic stress but not ionic stress. No remarkable differences was found between the *Oshak12* mutants and wild type plants (Supplementary Figures 4A–C). These results showed that the salt hypersensitivity of the *Oshak12* mutants probably due to Na^+^ ionic toxicity but not to osmotic damage.

**FIGURE 4:**
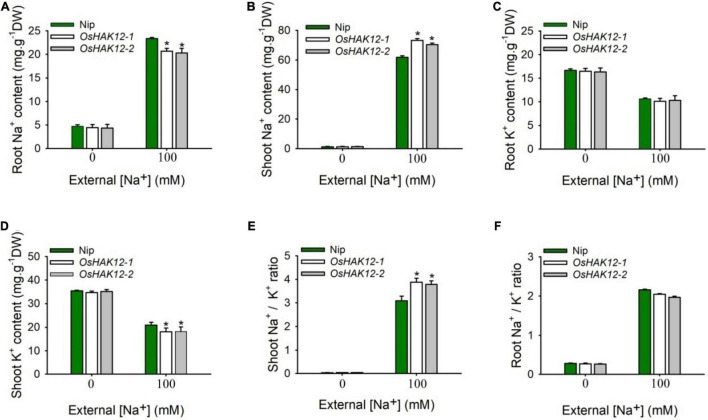
Disruption of *OsHAK12* affects Na^+^ and K^+^ ionic accumulation during salt stress. Na^+^ and K^+^ contents of the Nip and *Oshak12* mutants (*Oshak12-1*, *Oshak12-2*) were measured by ICP-MS. Growth conditions were as described in Figure 1A. **(A)** Root Na^+^ content of the Nip and *Oshak12* mutants. Significant differences were found between the Nip and *Oshak12* mutants (*n* = 50 for each data point) (*P < 0.005 by Student’s *t*-test). **(B)** Shoot Na^+^ content of the Nip and *Oshak12* mutants. Significant differences were found between the Nip and *Oshak12* mutants (*n* = 50 for each data point) (*P < 0.005 by Student’s *t*-test). **(C)** Root K^+^ content of the Nip and *Oshak12* mutants. No significant differences were found between the Nip and *Oshak12* mutants (*n* = 50 for each data point) (*P* > 0.05 by Student’s *t*-test). **(D)** Shoot K^+^ content of the Nip and *Oshak12* mutants. Significant differences were found between the Nip and *Oshak12* mutants plants (*n* = 50 for each data point) (*P < 0.01 by Student’s *t*-test). **(E)** Shoot Na^+/^K^+^ ratio in Nip and *Oshak12* mutants. The Nip and *Oshak12* mutants showed significant differences (*P < 0.01 by Student’s *t*-test). **(F)** Root Na^+/^K^+^ ratio in Nip and *Oshak12* mutants. The Nip and *Oshak12* mutants showed no significant differences (*P* > 0.05 by Student’s *t*-test). The experiment was repeated three times with similar results. Data are means of three replicates of one experiment. Asterisks represent significant differences. Error bars represent ± SD.

We then examined the Na^+^ contents in both shoot and root tissues of the above different genotypes plants during different NaCl concentrations. Under control condition (0 mM Na^+^), we found no significant differences of Na^+^ contents in roots or shoots between the mutants and wild type plants. However, under saline condition (100 mM Na^+^), *Oshak12* mutant plants contained significantly higher levels of Na^+^ in their shoots but lower levels of Na^+^ in their roots as compared with the wild type plants (Figures 4A,B). These above results suggested that knockout of *OsHAK12* leads to excessive root-to-shoot Na^+^ translocation in rice plants, resulting in over accumulation shoot Na^+^. Meanwhile, *Oshak12* mutant plants had significantly less shoot K^+^ and similar root K^+^ content compared with wild-type plants under saline condition (Figures 4C,D). As a result, the *Oshak12* mutants showed higher Na^+^/K^+^ ionic content ratio in shoots and similar Na^+^/K^+^ ionic content ratio in roots compared to those ratios in wild type plants (Figures 4E,F), which indicate that disruption of *OsHAK12* damaged the Na^+^/K^+^ ionic homeostasis in shoots during salt stress.

### *Oshak12* Mutants Show Less Na^+^ Retrieval From the Xylem in the Root

The expression analysis suggested that *OsHAK12* showed strong expression in root vascular tissues including xylem parenchyma cells (Figure 2Cii). Direct Na^+^ measurements suggested that, under saline conditions, the *Oshak12* mutants accumulated more Na^+^ in the shoot and less Na^+^ in the root than wild type plants (Figures 4A,B). These results indicate that *OsHAK12* may be involved in Na^+^ retrieval from the xylem vessels to xylem parenchyma cells in root tissues to prevent root-to-shoot Na^+^ translocation.

**FIGURE 5:**
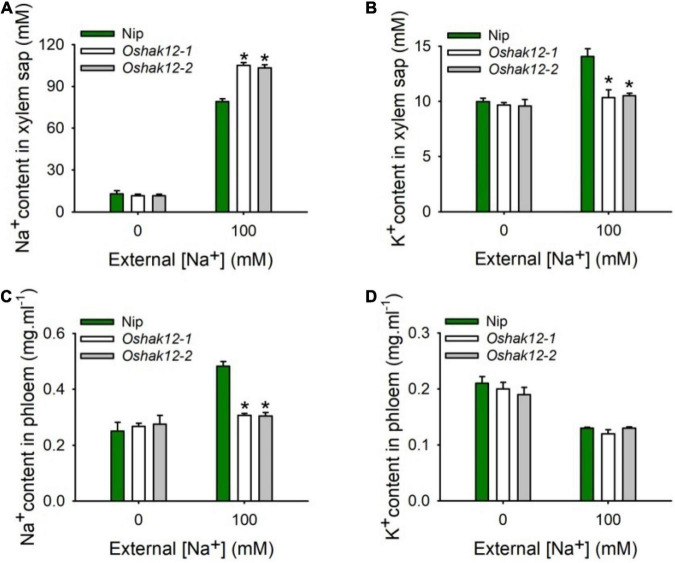
Effects of *Oshak12* disruption on Na^+^ and K^+^ ionic contents in xylem sap and phloem sap under salt stress. **(A)** Na^+^ content in xylem sap. 5-days-old rice seedlings were cultivated in the solutions for 14 days and then transferred to the hydroponic cultures containing 0 or 100 mM Na^+^ for 2 days. The Nip and *Oshak12* mutants (*Oshak12-1*, *Oshak12-2*) plants showed significant differences (*n* = 30 for each data point) (*P < 0.005 by Student’s *t*-test). **(B)** K^+^ concentration in xylem sap. Cultivation conditions were as described in **(A)**. The Nip and *Oshak12* mutants plants showed significant differences (*n* = 30 for each data point) (*P < 0.01 by Student’s *t*-test). **(C)** Na^+^ concentration in phloem. Growth conditions were as described in **(A)**. Na^+^ concentration were examined by ICP-MS. The Nip and *Oshak12* mutants plants showed significant differences (*n* = 30 for each data point) (*P < 0.005 by Student’s *t*-test). **(D)** K^+^ concentration in phloem. Growth conditions were as described in **(A)**. K^+^ concentration were examined by ICP-MS. The Nip and *Oshak12* mutants plants showed no significant differences (*n* = 30 for each data point) (*P* > 0.05 by Student’s *t*-test). The methods for the shoot excision, collecting the xylem sap and phloem secretion, examining Na^+^ and K^+^ concentration by ICP-MS were described previously by Tian et al. (2021). The experiment was repeated four times with similar results. Data are means of 20 replicates of one experiment. Asterisks represent significant differences. Error bars represent ± SD.

To address the role of OsHAK12 in regulating Na^+^ retrieving from the xylem sap to xylem parenchyma cells, the Na^+^ content in the xylem sap from different plants were measured. We found that, under control condition, there was no significant difference on Na^+^ content in the xylem vessels between the *Oshak12* mutants and wild type plants. However, under 100 mM NaCl, the *Oshak12* mutant plants had a significantly higher Na^+^ content in the xylem vessels than the wild type (Figure 5A), indicating that Na^+^ retrieving from the xylem vessels was defective in *Oshak12* mutants root tissues. Meanwhile, we observed reduced K^+^ content in the xylem sap, reduced Na^+^ content and similar K^+^ content in the phloem sap in the *Oshak12* mutant plants as compared with wild type plants (Figures 5B–D), which suggest that knockout of *OsHAK12* also affect Na^+^ loading into the phloem sap and K^+^ homeostasis in the xylem sap. Considering that OsHAK12 is barely expressed in the phloem tissues (Figure 2Ciii), the role of OsHAK12 in phloem needs further investigation.

Taken together, our data suggest that OsHAK12 mediates Na^+^ retrieving from the xylem vessels to xylem parenchyma cells, then decrease the Na^+^ content in the xylem sap, therefore reducing Na^+^ translocation from root to shoot, eventually promotes shoot Na^+^ exclusion under high salt conditions.

### *OsHAK12* Encodes a Na^+^- Permeable Transporter

Many HAK transporters display K^+^-transporting activity, however, some of HAK members were permeable to Na^+^ (Benito et al., 2012; Zhang et al., 2019). To evaluate the ion transport properties of OsHAK12 and interpret its vivo function under salt stress, we expressed OsHAK12 in the K^+^ uptake-deficient yeast strain CY162 (*MAT*α, Δ*trk1*, *trk2*:pCK64, *his3*, *leu2*, *ura3*, *trp1*, and *ade2*) (Anderson et al., 1992) and Na^+^ sensitive yeast strain AXT3K (Δ*ena1*:*HIS3*:*ena4*, Δ*nha1*:*LEU2*, Δ*nhx1*:*KanMX4*) (Quintero et al., 2002), respectively, and examined the effect of OsHAK12 expression on the growth of these yeast strains under different ionic conditions.

**FIGURE 6:**
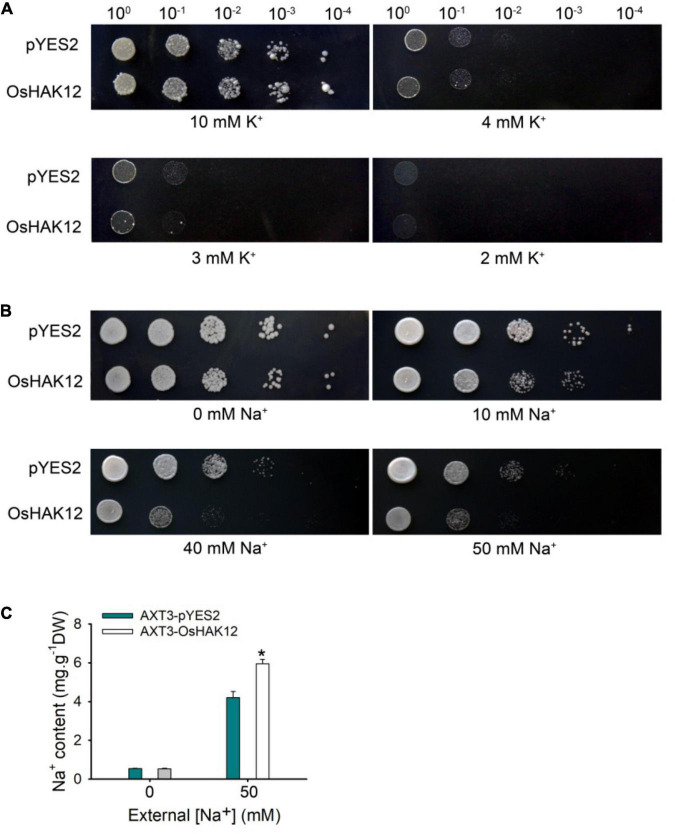
Functional complementation analysis of OsHAK12 in yeast mutants. **(A)** The empty vector pYES2 and pYES2 -OsHAK12 were separately introduced into the K^+^ uptake deficient yeast strain CY162. The overnight cultures were harvested and the optical density at 600 nm were adjusted to 1.0, then cultured on the AP medium containing 2, 3, 4, or 10 mM K^+^ at 30°C for 4 days. No significant differences were found between pYES2 and OsHAK12 when grown on the AP medium containing different K^+^ concentrations. **(B)** The empty vector pYES2 and pYES2 -OsHAK12 were separately introduced into the Na^+^ sensitive yeast strain AXT3K. The overnight cultures were harvested and the optical density at 600 nm were adjusted to 1.0, then cultured on the AP medium containing 0, 10, 40, or 50 mM Na^+^ at 30°C for 4 days. Significant differences were found between pYES2 and OsHAK12 when grown on the AP medium above 10 mM Na^+^. **(C)** Na^+^ concentration in AXT3K cells expressing the empty vector pYES2 and pYES2-OsHAK12. The Na^+^ concentration in AXT3K cells expressing the empty vector pYES2 and pYES2 -OsHAK12 showed significant differences (*P < 0.01 by Student’s *t*-test). The experiment was repeated five times with similar results. Data are means of three replicates of one experiment. Asterisks represent significant differences. Error bars represent ± SD.

The yeast strain CY162 lacking the high-affinity K^+^-transporters Trk1/2, therefore defective in K^+^ uptake (Anderson et al., 1992). We expressed OsHAK12 in the yeast strain CY162 to determine whether OsHAK12 can mediate K^+^ transport. When grown on the arginine phosphate (AP) medium containing 10 mM K^+^, yeast strain CY162 grew well with or without *OsHAK12* construct (Figure 6A). A similar growth was also observed under 3 mM K^+^ (Figure 6A). When the K^+^ concentration in the AP medium was lower to less than 3 mM, yeast mutant transformed with either the empty vector or *OsHAK12* construct both failed to grow on AP medium (Figure 6A). The results indicated that OsHAK12 confers little K^+^-transporting activity. The yeast complementation data were consistent with the finding that disruption of *OsHAK12* did not affect K^+^ homeostasis in rice plants under non-saline conditions (Supplementary Figures 2, 3).

The *Saccharonmyces cerevisiae* strain AXT3K lacking major Na^+^ transporters (Na^+^ efflux proteins ENA1-4 and NHA1, and the vacuolar Na^+^/H^+^ antiporter NAX1) crucial for high-Na^+^ tolerance of yeast, which revealed growth inhibition above 50 mM Na^+^ concentrations (Quintero et al., 2002), so we expressed OsHAK12 in the yeast strain AXT3K to determine whether OsHAK12 can mediate Na^+^ influx. When grown on AP medium without Na^+^, Both transformants harboring either the empty vector or *OsHAK12* construct showed similar growth (Figure 6B). However, when the Na^+^ concentration in AP medium was increased to 10 mM, yeast mutant transformed with OsHAK12 construct showed a hypersensitive phenotype than that of the empty vector control, and this phenotype became more evident when the Na^+^ concentration in AP medium was increased to 50 mM (Figure 6B), indicating OsHAK12 confers Na^+^-transporting activity. To verify this result, we then examined Na^+^ contents in the transformed AXT3K yeast strains, the results showed that *OsHAK12*-expressing AXT3K cells accumulated more Na^+^ content than that in the controls when exposed to 50 mM NaCl (Figure 6C). These observations indicate that OsHAK12 can mediate Na^+^ transport. Considering its expression pattern (mainly in roots) and subcellular localization (in plasma membrane), its disruption was responsible for the hypersensitivity to salinity stress and functions in Na^+^ retrieving from the xylem vessels (Figures 1–3, 5A), we suggest that OsHAK12, may be as a Na^+^-permeable transporter mediating Na^+^ transport in rice roots.

## Discussion

Salt tolerance is developing as an significant agronomical trait of crop breeding. Na^+^ exclusion from shoot is vital for plants adaption to high salt environments (Munns and Tester, 2008; Ismail and Horie, 2017; Zelm et al., 2020). Here, we display that *OsHAK12* functions as a Na^+^- permeable plasma membrane transporter, mediating Na^+^ retrieving from the xylem vessels back to root tissues, then promoting shoot Na^+^ exclusion, thus safeguarding plant shoots from salt toxicity.

Na^+^ is excluded from shoots, meanwhile K^+^ is accumulated in shoots, thus keeping the high cytosolic K^+^/Na^+^ ionic content ratio in shoots during salt toxicity (Ren et al., 2005; Ismail and Horie, 2017). Therefore maintenance of cytoplasm K^+^/Na^+^ ionic homeostasis is tightly linked with the salt tolerance in plant, which depends on the directions of the plasma membrane K^+^/Na^+^ transporters (Ren et al., 2005; Ismail and Horie, 2017; Zelm et al., 2020). Previous studies displayed that high affinity K^+^ transporters (HAKs) play essential roles in maintaining K^+^/Na^+^ homeostasis in rice under salt stress (Horie et al., 2011; Ismail and Horie, 2017). For example, the disruption of *OsHAK5* was responsible for the hypersensitivity to salinity stress and lower shoots K^+^/Na^+^ ionic content ratio. It elevates shoots K^+^/Na^+^ ionic content ratio by increasing root K^+^ uptake and root-to-shoot K^+^ translocation, then improved rice salt tolerance during salt stress (Yang et al., 2014). K^+^ uptake was almost completely damaged by the disruption of *OsHAK1* in rice under the salt stress, thus the plants displayed lower K^+^/Na^+^ ionic content ratio in both roots and shoots and led to sensitivity to salt stress (Chen et al., 2015). OsHAK16 and OsHAK21 also increasing K^+^/Na^+^ ionic content ratio in shoot by enhancing K^+^ uptake in root, thus maintain salt tolerance in rice (Shen et al., 2015; Feng et al., 2019). The above studies showed that K^+^ uptake in root display important roles on K^+^/Na^+^ ionic homeostasis and salt tolerance in plants. Here, we show that OsHAK12 maintains K^+^/Na^+^ ionic homeostasis and salt tolerance in rice during salt stress by retrieving Na^+^ from the xylem vessel, which is different from the above reported OsHAKs -mediated mechanism in rice salt tolerance, suggesting that OsHAK12 regulate salt tolerance in a novel manner.

Ion transport properties assays show that reported OsHAK members as K^+^-selective transporters maintain rice salt tolerance (Yang et al., 2014; Chen et al., 2015; Feng et al., 2019). For instance, OsHAK5, OsHAK16, and OsHAK21 were reported to complement the growth defects of the K^+^ uptake-deficient yeast mutant CY162 and R5421 but not the Na^+^ exclusion-deficient *E. coli* mutant strain KNabc and yeast strain G19, respectively (Horie et al., 2011; Yang et al., 2014; Shen et al., 2015; Feng et al., 2019). In addition, expression of OsHAK1, OsHAK5, OsHAK16, and OsHAK21 in the K^+^ uptake-deficient yeast strain CY162 all increase their salt tolerance by mediating cellular K^+^ uptake (Yang et al., 2014; Chen et al., 2015; Shen et al., 2015; Feng et al., 2019). The above complementation assay in the yeasts or E. coli both demonstrated that reported OsHAKs all are as K^+^-selective transporters to maintain cell salt tolerance. However, OsHAK12 displays Na^+^-transporting activity to confer cell salt tolerance using yeast complementation systems. All of above datas show that unlike reported OsHAKs, OsHAK12 serves as a Na^+^-permeable transporter to confer salt tolerance by mediating Na^+^ transport in rice roots. However, whether other OsHAK transporters as Na^+^- permeable transporter confer salt tolerance in rice remain an open question.

Interestingly, studies have recently highlighted the impact of a Na^+^-selective HAK family member ZmHAK4-mediated Na^+^ exclusion from shoot on the salt tolerance in maize (Zhang et al., 2019). ZmHAK4 is a Na^+^-selective transporter, which probably promotes shoot Na^+^ exclusion and salt tolerance by retrieving Na^+^ from xylem vessel (Zhang et al., 2019). These datas suggest that OsHAK12 and ZmHAK4 mediate shoot Na^+^ exclusion in monocot crop plants in a similar manner, which also addressing *HAK*-type transporters probably confer a conserved mechanism against salinity stress in monocot crops. However, there are also exist some different transport properties between OsHAK12 and ZmHAK4. For example, disruption of OsHAK12 and ZmHAK4 led to different defects of Na^+^ exclusion from shoot, with *Zmhak4* mutants showing defects during the conditions with Na^+^ concentrations ranging from submillimolar levels to over 100 mM (Zhang et al., 2019), whereas *Oshak12* mutants showing defects only under high-Na^+^ conditions (Figure 1). These observations indicate that OsHAK12 and ZmHAK4 may confer different roles to ensure shoot Na^+^ exclusion.

Geography and rainfall variation lead to fluctuating Na^+^ concentrations in soil. Thus, plants need precise control processes to achieve Na^+^ homeostasis in response to salt stress (Ismail and Horie, 2017; Zelm et al., 2020). Previous study showed that rice Na^+^ transporter OsHKT1;5 also prevent shoot Na^+^ overaccumulation by mediating Na^+^ exclusion from xylem sap thereby safeguarding leaves from salinity toxicity (Ren et al., 2005). Our datas showed that OsHAK12-mediated Na^+^ exclusion from xylem vessels involve a similar mechanism as OsHKT1;5. It is noticeable that the *OsHAK12* expression pattern has some difference compare with that of *OsHKT1;5*. For instance, the expression of *OsHKT1;5* was present predominately in the vascular tissues of various organs, such as roots, leaves, leaf sheath bases, nodes and internodes (Ren et al., 2005), whereas *OsHAK12* was expressed mainly in root vascular tissues (Figure 2C). Studies also showed that OsHKT1;5 mediates xylem Na^+^ unloading from leaf sheaths phloem in rice, which prevents Na^+^ transfer to young leaf blades, then protect leaf blades from salt toxicity (Kobayashi et al., 2017). However, whether OsHAK12 is involved in these processes remain unknown. These observations indicate that OsHAK12 and OsHKT1;5 may confer different roles or work together to ensure the precise control of Na^+^ exclusion from shoot. This hypothesis should be investigated by future experiments. Previous studies showed that the first glycine/serine residue in the first P-loop in OsHKT1 and OsHKT2 protein struct is crucial for K^+^/Na^+^ selectivity and transport (Mäser et al., 2002; Ródenas et al., 2021), however, the K^+^ selective filter motif was lacked and mutated in OsHAK12 and OsHKT1;5 protein structures, respectively, which may further suggest OsHAK12 and OsHKT1;5 both are Na^+^ permeable-transporters (Supplementary Figures 5, 6). In addition, whether mutation in other positions in the genomic of *OsHAK12* affect the phenotype under salt stress need to be further investigated. Consequently, understanding the molecular interaction among the individual HAK transporters and other Na^+^ transport family members in rice will provide a helpful platform for breeding salt tolerance rice varieties.

## Data Availability Statement

The original contributions presented in the study are included in the article/Supplementary Material, further inquiries can be directed to the corresponding author/s.

## Author Contributions

LZ, DM, LC, and SL conceived, designed the experiments, and analyzed the data. LZ, XS, YL, YC, SS, and XW performed the experiments. LZ, DM, and SL wrote the manuscript. All authors contributed to the article and approved the submitted version.

## Conflict of Interest

The authors declare that the research was conducted in the absence of any commercial or financial relationships that could be construed as a potential conflict of interest.

## Publisher’s Note

All claims expressed in this article are solely those of the authors and do not necessarily represent those of their affiliated organizations, or those of the publisher, the editors and the reviewers. Any product that may be evaluated in this article, or claim that may be made by its manufacturer, is not guaranteed or endorsed by the publisher.

## Footnotes

1
http://cbi.hzau.edu.cn/cgi-bin/CRISPR
