# Supplementary material for: Rice Na+-Permeable Transporter OsHAK12 Mediates Shoots Na+ Exclusion in Response to Salt Stress
Source: Front Plant Sci. 2021 Dec 7;12:771746. doi: 10.3389/fpls.2021.771746 (PMC8688356; doi:10.3389/fpls.2021.771746)
Supplement: Supplementary file 1 [file Data_Sheet_1.pdf]

Supplemental datas

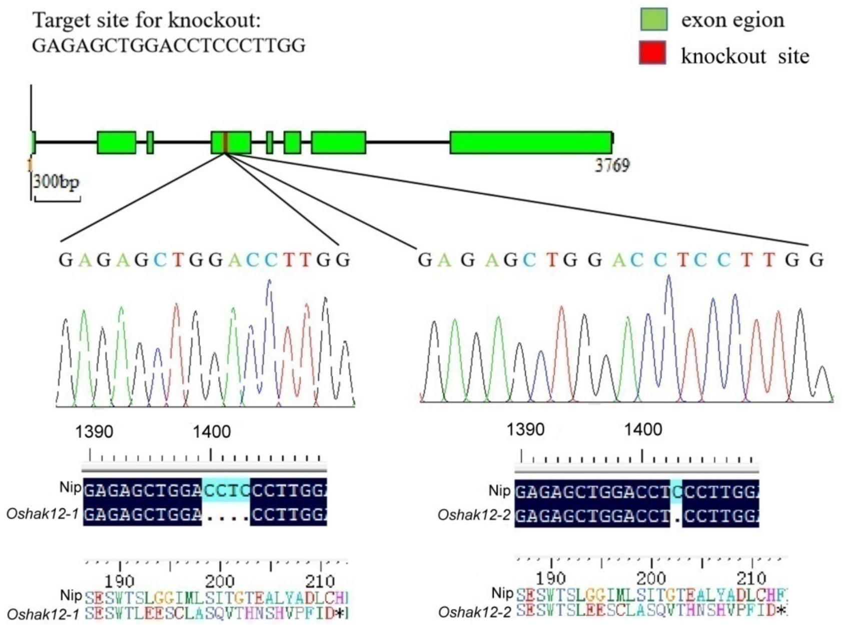

Figure S1. Confirmation of the *Oshak12* mutants.

PCR-based sequencing was used to verify the *Oshak12* mutants. Two independent transgenic lines (*Oshak12-1* and *Oshak12-2*) for *Oshak12* mutants are gained from *Nipponbare* rice background.

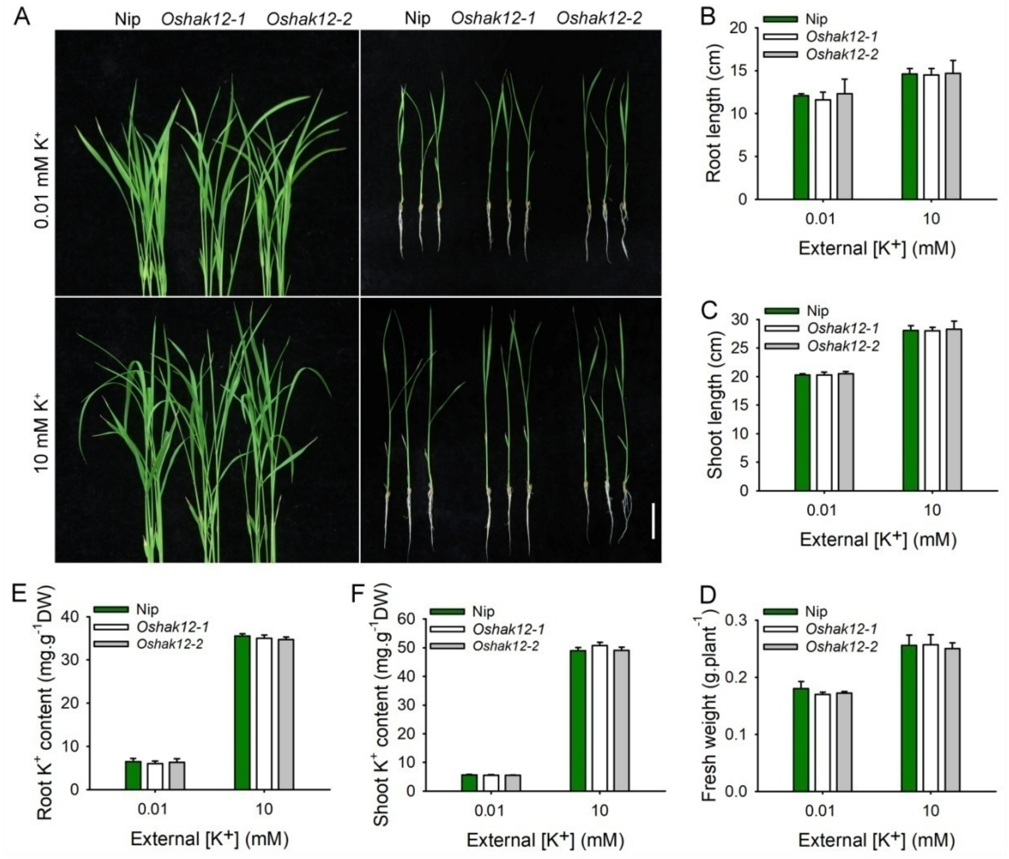

Figure S2. ***Oshak12*** **mutants are not sensitive to different K^+ concentrations treatments.**

(A) *Oshak12* mutants are not sensitive to different K^+ concentrations treatment. 5-d-old rice seedlings of the Nip and *Oshak12* mutants (*Oshak12-1*, *Oshak12-2*) were cultivated in hydroponic cultures with 0.01 or 10 mM K^+ for 14 d, respectively. The growth of the *Oshak12* mutants (*Oshak12-1*, *Oshak12-2*) showed no discernible differences compared with Nip plants under different K^+ concentration conditions. Bars = 4 cm.

(B) Root length of the Nip and *Oshak12* mutants plants.

(C) Shoot length of the Nip and *Oshak12* mutants plants.

(D) Fresh weight of Nip and *Oshak12* mutants plants.

(E) Root K^+ content of Nip and *Oshak12* mutants plants.

(F) Shoot K^+ content of Nip and *Oshak12* mutants plants.

No significant differences were found between the Nip and *Oshak12* mutants (n = 50 for each data point) ($P > 0.05$ by Student's t test). Growth conditions were as described in Figure S2A. The experiment was repeated four times with similar results. Data are means of five replicates of one experiment. Error bars represent ±SD.

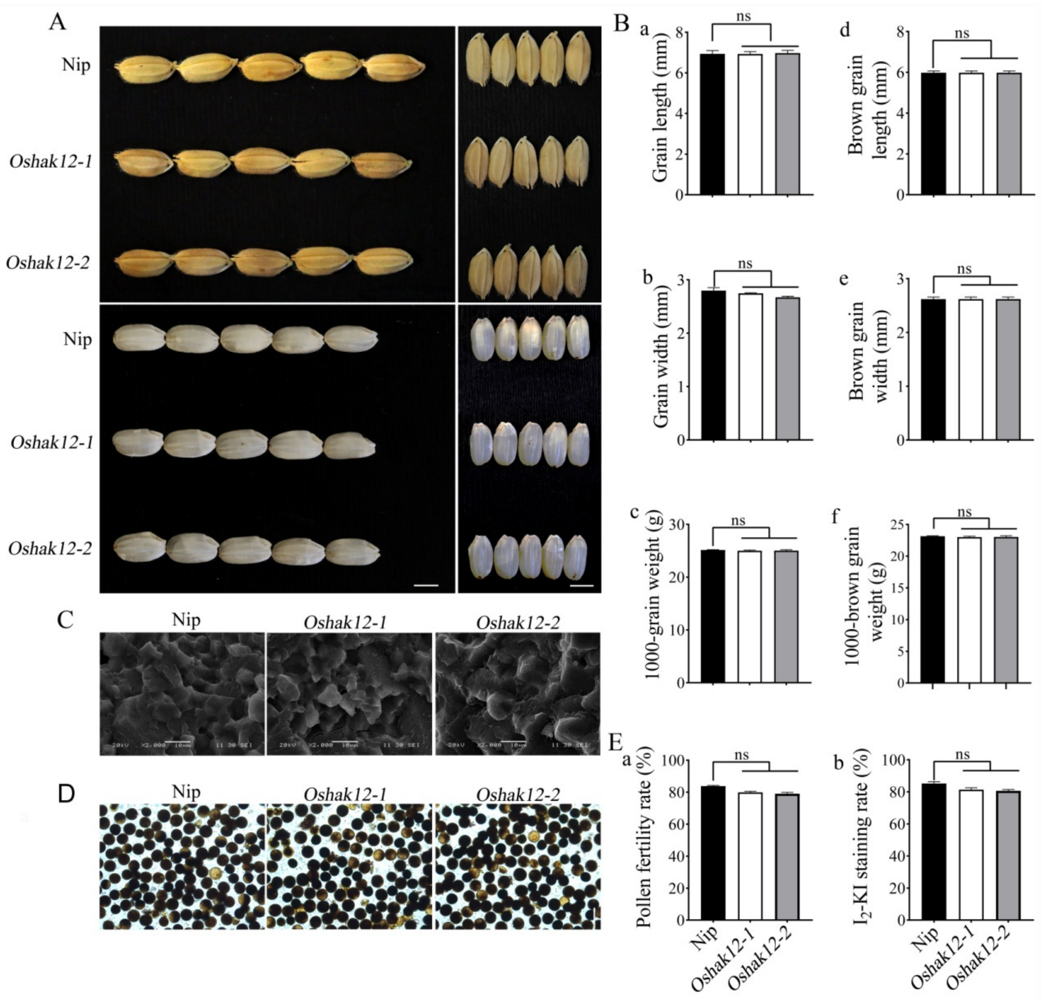

Figure S3. Grain morphology features of *Oshak12* mature grains.

(A) Grains phenotypic analysis of *Oshak12* mutants. Grain length (left) and grain width (right) of the mature grains (upper) and brown grain (lower) (after DAF 43) of Nip and *Oshak12* mutants (*Oshak12-1*, *Oshak12-2*). Images shown were digitally extracted and scaled for comparison. Bars = 1 cm.

(B) Statistical data of grains of Nip and the *oshak12* mutant. Statistical data of grain length (a), grain width (b), 1000-grain weight (c), brown grain length (d), brown grain width (e), and brown 1000-grain weight (f). n = 50 for (a-c) and 1000 for (d, f).

(C) Scanning electron microscope images of transverse sections of starch grains in the endosperm of the Nip and *Oshak12* mature brown grains as described in Figure S3A. Bar = 10 μm.

(D) Staining of pollen grains by I_2-KI from Nip and *Oshak12* mutants. Bar = 100μm.

(E) Pollen viability of Nip and *Oshak12* mutants. Pollen fertility rate (a) and I_2-KI staining rate (b) of Nip and *Oshak12* mutants.

The Nip and *Oshak12* mutants plants showed no significant difference ($P > 0.05$ by Student's t test). The experiment was repeated five times with similar results. Error bars represent ±SD.

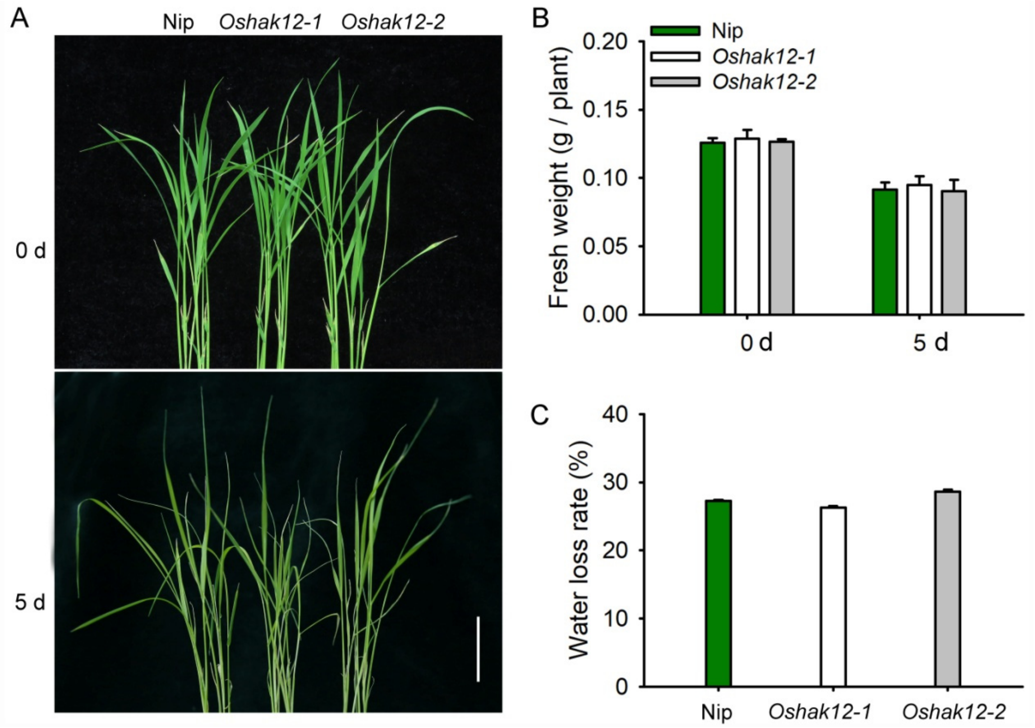

Figure S4. ***Oshak12*** **mutants are not sensitive to PEG treatment.**

(A) *Oshak12* mutants are insensitive to PEG treatment. 5-d-old rice seedlings of the Nip and *Oshak12* mutants (*Oshak12-1, Oshak12-2*) were transferred to the hydroponic cultures for 14 d, respectively, then transferred to hydroponic solution containing 20% PEG and photographed after 5 d. Bars = 6 cm.

(B) Fresh weight of the Nip and *Oshak12* mutants under 20% PEG treatment. Growth conditions were as described in Figure S4A.

(C) Water loss rate of the Nip and *Oshak12* mutants under 20% PEG treatment. Growth conditions were as described in Figure S4A.

The Nip and *Oshak12* mutants plants showed no significant differences (P > 0.05 by Student's t test). The experiment was repeated four times with similar results. Data are means of five replicates of one experiment. Error bars represent ±SD.

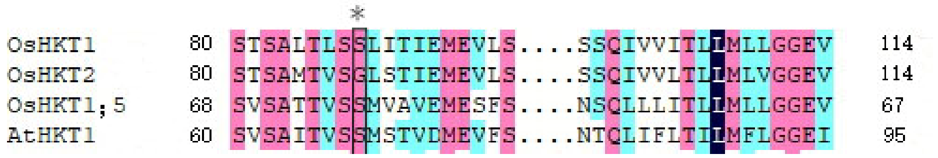

Figure S5. Alignment of the first P-loop A conserved sequences of related HKT proteins

Alignment of the conserved sequences in the first P-loop A of OsHKT1, OsHKT2, OsHKT1;5, and AtHKT1.

The first glycine of the K^+ selectivity filter GYG motif is marked with an asterisk.

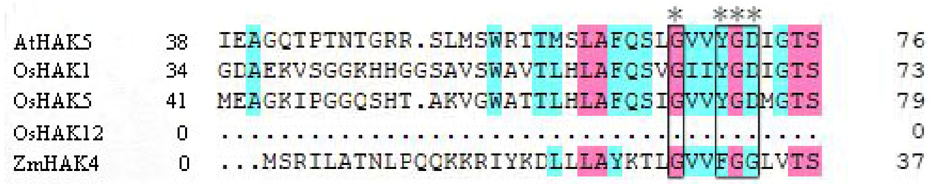

Figure S6. Alignment of the conserved sequences in the TM1,TM3, and TM6 of related HAK proteins

Alignment of the conserved sequences in the TM1,TM3, and TM6 of AtHAK5, OsHAK1, OsHAK5, OsHAK12, and ZmHAK4. Conserved residues with the K^+ selectivity filter are marked with asterisks.

Table S1. List of PCR Primers

Name	Primer sequences	Purposes
OsHAK12-Cas9-F2	ggcaGAGAGCTGGACCTCCCTTGG	CRISPR/Cas9 construction
OsHAK12-Cas9-R2	aaacCCAAGGGAGGTCCAGCTCTC	
OsHAK12-T1-F	TTCAGAGCCTTGGTGTTG	CRISPR/Cas9 positive determination
OsHAK12-T1-R	AATGTTCTGTTATTTATGTGCC	
OsHAK12-CDS-F	ATGAGTACAGATGTGGTTGTAGTCGTT	Cloning of *OsHAK12* CDS sequence
OsAK12-CDS-R	TCATATATAGTATATCTGGCCTACATTGAGA	
OsHAK12-pCAMBIA1301-F	tatgaccatgattacgaattcGTTGAGCAAATGAATGCTTCA TTT	Cloning of *OsHAK12* promoter sequence
OsHAK12-pCAMBIA1301-R	acgacggccagtgccaagcttATCAGAGGAATGAGGGTGA GGG	
OsSP1-pCAMBIA1300-RFP-F	ggtacccggggatcctctagaATGGATGTTGAGTCAAGGC	Subcellular localization construction
OsSP1-pCAMBIA1300-RFP-R	gctcaccatgtcgactctagaGTGACCCATGTTGACCTCGT	
OsHAK12-pCAMBIA1390-F	gacagggtacccggggatcc ATGAGTACAGATGTGGTTAT	Subcellular localization construction
OsHAK12-pCAMBIA1390-R	agctcctcctcctcctctaga TATATAGTAT ATCTGGCCTA	
OsHAK12-PYES2-F	actatagggaatattaagcttATGAGTACAGATGTGGTTGTA GTCGTT	CY162 complementation construct of *OsHAK12*
OsAK12-PYES2-R	tacatgatgcggccctctagaTCATATATAGTATATCTGGCCT ACATTG	
RT-*OsHAK12*-F	CGTCGTCTTCGTTTGTGTCSSG	Q-PCR analysis of *OsHAK12* expression pattern
RT-*OsHAK12*-R	CTTTGGCCCGATCCTCTTC	
OsActin-F	CAATGTGCCAGCTATGTATGTCGCC	Q-PCR analysis of *OsActin*
OsActin-R	TTCCCGTTCAGCAGTGGTAGTGAAG	
